# Changes in Cortical Thickness in Patients With Early Parkinson’s Disease at Different Hoehn and Yahr Stages

**DOI:** 10.3389/fnhum.2018.00469

**Published:** 2018-11-27

**Authors:** Yuyuan Gao, Kun Nie, Mingjin Mei, Manli Guo, Zhiheng Huang, Limin Wang, Jiehao Zhao, Biao Huang, Yuhu Zhang, Lijuan Wang

**Affiliations:** ^1^Department of Neurology, Guangdong General Hospital, Guangdong Academy of Medical Sciences, Guangdong Neuroscience Institute, Guangzhou, China; ^2^Department of Radiology, Guangdong General Hospital, Guangdong Academy of Medical Sciences, Guangzhou, China

**Keywords:** Parkinson’s disease, Hoehn and Yahr stage, UPDRS part III, FreeSurfer, magnetic resonance imaging

## Abstract

**Objectives:** This study was designed to explore changes in cortical thickness in patients with early Parkinson’s disease (PD) at different Hoehn and Yahr (H-Y) stages and to demonstrate the association of abnormally altered brain regions with part III of the Unified Parkinson’s Disease Rating Scale (UPDRS-III).

**Materials and Methods:** Sixty early PD patients and 29 age- and gender-matched healthy controls (HCs) were enrolled in this study. All PD patients underwent comprehensive clinical and neuropsychological evaluations and 3.0 T magnetic resonance scanning. Patients with H-Y stage ≤1.5 were included in the mild group, and all other patients were included in the moderate group. FreeSurfer software was used to calculate cortical thickness. We assessed the relationship between UPDRS-III and regional changes in cortical thinning, including the bilateral fusiform and the temporal lobe.

**Results:** The average cortical thickness of the temporal pole, fusiform gyrus, insula of the left hemisphere and fusiform gyrus, isthmus cingulate cortex, inferior temporal gyrus, middle temporal cortex and posterior cingulate cortex of the right hemisphere exhibited significant decreasing trends in HCs group and PD groups (i.e., the mild group and moderate group). After controlling for the effects of age, gender, and disease duration, the UPDRS-III scores in patients with early PD were correlated with the cortical thickness of the left and right fusiform gyrus and the left temporal pole (*p* < 0.05).

**Conclusion:** The average cortical thickness of specific brain regions reduced with increasing disease severity in early PD patients at different H-Y stages, and the UPDRS-III scores of early PD patients were correlated with cortical thickness of the bilateral fusiform gyrus and the left temporal pole.

## Introduction

Parkinson’s disease is the second most common neurodegenerative disease, next only to AD, and is characterized by motor symptoms, such as bradykinesia, rest tremor and muscle rigidity, and non-motor symptoms (NMS), such as cognitive impairment, constipation, anxiety, depression, and sleep disorders. The main pathological changes in PD are the loss of large quantities of dopaminergic neurons from the midbrain substantia nigra pars compacta and the formation of Lewy bodies by misfolded α-synuclein, resulting in basal ganglia dysfunction (Kalia and Lang, 2015). The prevalence of PD is 1–2% among elderly over 65 years of age and 3–5% in those over 85 years of age (Alves et al., 2008). By 2030, there are expected to be five million PD patients in China (Dorsey et al., 2006). Two hundred years after the first documented description of PD (Kalia and Lang, 2015) and although the core pathological features of PD have been established, the related pathogenic mechanism of the misfolded α-synuclein remains controversial (Kalaitzakis et al., 2008). In 2003, Braak et al. (2003) divided PD into six stages to describe the development of PD (Braak et al., 2003). According to Braak’s staging system, the evolution of motor symptoms and NMS of PD can be well explained. The onset of motor symptoms is suggested to accompany the death of at least 50% of the dopaminergic neurons in the substantia nigra, corresponding to Braak stage III/IV. However, patients at Braak stages I and II mainly exhibit NMS, such as orthostatic hypotension, olfactory dysfunction and rapid eye movement (REM) sleep abnormalities. Therefore, early identification of preclinical and early-stage PD patients may aid in the early management of PD.

The Hoehn-Yahr (H-Y) scale was created in 1967 and is widely used in the evaluation of the clinical condition of PD (Hoehn and Yahr, 1967). The quality of life of PD patients is significantly reduced during disease development, whereby patients progress from modified H-Y stage ≤2.5 to modified H-Y stage >3 (Evans et al., 2011). The guidelines for the diagnosis of PD in China define patients with H-Y stage ≤2.5 as early PD patients (Chen et al., 2016). PD patients at different stages present brain structural changes, but the related pathogenic mechanism and pathophysiological changes remain unclear. The preclinical stage of PD is very difficult to diagnose because the symptoms and signs of patients are not typical, and there are no significant changes in anatomical images, such as CT and MRI. However, PD patients gradually present clinical symptoms and signs with increasing H-Y stages and exhibit the typical symptoms at middle and late stages, which are not difficult to diagnose. Therefore, the identification of early PD imaging signs is of great importance for the early diagnosis of PD. Changes in cortical thickness (Mak et al., 2015), GM volume (Jia et al., 2015) and cortical microstructure (Nurnberger et al., 2017), increased cortical and subcortical atrophy rate (Tessa et al., 2014) and loss of cortical gyrification (Sterling et al., 2016) have been reported, and some imaging signs even correlate with disease progression (Ibarretxe-Bilbao et al., 2011; Jubault et al., 2011). Specifically, cortical thickness can be used in PD disease staging and cognitive impairment assessment (Zarei et al., 2013). Therefore, we hypothesized that abnormal changes in cortical thickness may occur in early-stage PD patients with H-Y stage ≤1.5 and H-Y stage ≥2 and that these changes would correlate with the severity of the disease and UPDRS-III. Therefore, this study investigated the cortical thickness abnormalities of early PD patients at different H-Y stages and the correlation between cortical thickness and UPDRS-III, providing imaging signs for the early identification of preclinical and early-stage PD patients.

## Materials and Methods

### Participants

**FIGURE 1:**
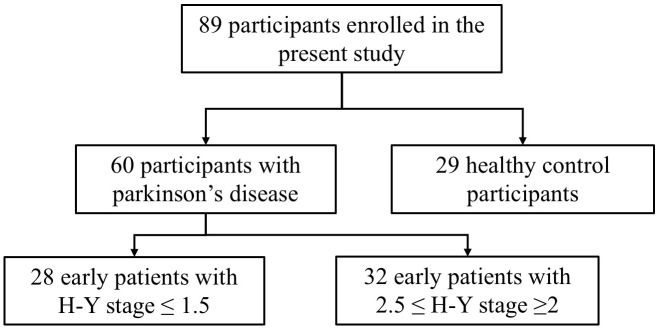
Flow chart of the study.

This study selected 89 subjects from a database of the Department of Neurology, Guangdong General Hospital (Guangzhou, China), including 60 patients with early stage PD and 29 gender-, age-, and education level-matched HCs. The diagnosis of PD was independently performed in accordance with the British Brain Bank PD diagnostic criteria by two neurologists who held positions of attending physicians. In addition, the PD patients underwent a series of assessments. The motor function in PD patients was assessed using UPDRS-III. The severity of PD was assessed using the H-Y stage. Depression in PD patients was evaluated using the 24-item HAMD. Anxiety in PD patients was assessed by the HAMA. The ADL of PD patients were evaluated using the 14-item ADL Scale. The cognitive function of PD patients was evaluated using the MoCA and the MMSE. All PD subjects included in this study met the following criteria: age ≥40 years, early stage PD (H-Y stage ≤2.5), and no severe depression or anxiety (HAMD ≤ 20 points and HAMA ≤ 29 points). According to the H-Y stage score, early PD patients with H-Y stage ≤1.5 were included in the mild group, and those with 2.5≤ H-Y stage ≥2 were included in the moderate group. Structural MRI was acquired from 28 early PD patients in the mild group and 32 early PD patients in the moderate group (Figure 1). Healthy volunteers did not have any neurological or mental illness and did not take medications known to affect the dopaminergic system. Structural MRI was acquired from 29 healthy subjects in the HC group. All subjects provided written informed consent to the research protocol, which was approved by the Medical Ethics Committee of Guangdong General Hospital.

### Collection of Magnetic Resonance Imaging (MRI) Data

A GE 3.0 T MR imaging system (GE Healthcare, Fairfield, United States) was used. The magnetic resonance data of all subjects were obtained by three-dimensional spoiled gradient recalled (3D-SPGR) sequences. During scanning, the subjects were positioned on a patient bed in the supine position. The head was fixed on the matrix coil with a sponge pillow and a helmet to minimize head movement, and earplugs were worn. High signal-to-noise ratio three-dimensional T1-weighted images were obtained by high-resolution scanning. The parameters were as follows: number of scanning slices, 146; slice thickness, 1.00 mm; slice spacing, 1.00 mm; matrix, 256 × 256; field of view (FOV), 512 × 512; voxel, 0.469 × 0.469 × 1.0 mm^3^; flip angle, 13°; repetition time (TR), 7.6 ms; and echo time (TE), 3.3 ms.

### Image Processing and Analysis

MRIcron software was used to convert the MRI data of all subjects from DICOM format to NIFTI format, and the process of extracting cerebral cortex thickness was based on FreeSurfer v5.3.0 software^1^ on the Linux platform (ubuntu 14.04) according to previously published papers (Fischl and Dale, 2000; Fischl, 2012). For image preprocessing, the “recon-all” instruction was used, which included magnetic field inhomogeneity correction, motion correction, removal of non-brain tissues such as the scalp and skull, segmentation of the GM and WM boundaries, automatic topological correction and registration with standard brain templates, Gaussian smoothing, reconstruction of the bilateral cerebral cortex, and calculation of the cortical thickness. MNI coordinates were transformed from the Talairach coordinates by using the tool provided in http://imaging.mrc-cbu.cam.ac.uk/downloads/MNI2tal/tal2mni.m.

### Statistical Analysis

SPSS 22.0 was used to analyze the data. A linear model (GLM) was defined for statistical analysis in the Qdec module of FreeSurfer. Brain areas with significant differences in cortical thickness, as determined by intergroup comparisons of the HC, mild and moderate groups, were identified. For the HC, mild and moderate groups, one-way ANOVA was used to compare age, the chi-square test was used to compare gender, and the Kruskal–Wallis *H* test was used to compare education. For the mild and moderate groups, disease duration, ADL, HAMA, MMSE and H-Y stage were compared using the Mann–Whitney *U* test, and UPDRS-III and MoCA were compared using a two-tailed *t*-test. Regression analysis of average cortical thickness of different brain areas and UPDRS-III scores using a general linear model while controlling for age, gender, and disease duration. A *p*-value <0.05 was considered statistically significant.

## Results

### Demographic and Clinical Characteristics of the HC, Mild and Moderate Groups

Among the three groups, no significant differences in age (*F* = 1.742, *p* = 0.181), gender (χ^2^ = 3.620, *p* = 0.164) and education (*H* = 0.048, *p* = 0.976) were noted. Comparison of the mild and moderate groups revealed no significant differences in disease duration (*Z* = -0.772, *p* = 0.440), ADL (*Z* = -1.243, *p* = 0.214), HAMD (*Z* = -1.889, *p* = 0.059), HAMA (*Z* = -1.176, *p* = 0.240), MoCA (*t* = -0.022, *p* = 0.983), MMSE (*Z* = -1.381, *p* = 0.167) and H-Y stage (*Z* = 6.864, *p* = 0.000), whereas significant differences in UPDRS-III (*t* = -4.847, *p* < 0.0001) were noted (Table 1).

**Table 1:** Demographic and clinical data of subjects in the HC, mild, and moderate groups.

Variables	HCs	Mild	Moderate	*F*/*H*/χ^2^/*t*	*p*-value
Subjects	29	28	32	–	–
Age in years, mean (SD)	59.17 (1.88)	57.32 (1.65)	61.59 (1.39)	1.742 (*F*)	0.181
Gender, male/female	12/17	17/11	12/20	3.620 (χ^2^)	0.164
Education in years, mean (SD)	9.69 (1.02)	10.04 (0.75)	9.81 (0.75)	0.048 (*H*)	0.976
Disease duration, month, mean (SD)	–	28.07 (3.34)	30.44 (6.37)	-0.772 (*Z*)	0.440
UPDRS-III, mean (SD)	–	19.29 (1.60)	31.08 (1.79)	-4.847 (*t*)	**<0.0001**
ADL	–	15.36 (0.58)	17.28 (0.97)	-1.243 (*Z*)	0.214
HAMD	–	4.93 (0.73)	7.41 (0.91)	-1.889 (*Z*)	0.059
HAMA	–	4.57 (0.71)	5.88 (0.75)	-1.176 (*Z*)	0.240
MoCA, mean (SD)	–	22.79 (0.93)	22.81 (0.82)	-0.022 (*t*)	0.983
MMSE, mean (SD)	–	28.14 (0.31)	27.25 (0.44)	-1.381	0.167
H-Y stage	–	1.5 (1.0–1.5)	2.25 (2.0–2.5)	6.864 (*Z*)	0.000

*PD patients with H-Y stage ≤1.5 were included in the mild group, and PD patients with 2.5≤ H-Y stage ≥2 were included in the moderate group. ADL, activities of daily living; UPDRS-III, part III of the Unified Parkinson’s Disease Rating Scale; HAMD, Hamilton Depression Scale; HAMA, Hamilton Anxiety Scale; MoCA, Montreal Cognitive Assessment; MMSE, Mini Mental State Examination; H-Y stage, Hoehn and Yahr stage. Significant value is indicated in bold*.

### Cortical Thickness Across the HC, Mild and Moderate Groups

The coordinates, number of vertices, maximum *p* value, extent of areas with differences in cortical thickness, and average cortical thicknesses of relevant brain areas can be found in Table 2.

**Table 2:** Anatomical location of brain areas exhibiting significant differences in intergroup comparisons of the HC, mild and moderate groups.

Contrast	Regions	P-Max	MNI coordinate			Total surface area size (mm^2^)	Vertices	Mean thickness (mm)		
			*X*	*Y*	*Z*			HCs	Mild	Moderate
Mild > Moderate *p*-value <0.001 Surface area size > 100	L temporal pole	4.3345	-34	10	-39	165.28	302	2.249	2.125	1.777
	L superior frontal cortex	4.0574	-14	20	37	453.91	946	1.919	1.923	1.803
	L insula	3.7611	-29	17	-8	455.60	1264	2.042	1.958	1.573
	L isthmus cingulate cortex	3.5572	-6	-53	29	135.91	315	2.173	2.039	1.929
	L fusiform gyrus	3.4439	-37	-53	-25	296.43	411	1.946	1.849	1.564
	R inferior temporal cortex	5.5224	52	-53	-22	332.07	574	2.101	2.088	1.833
	R lateral occipital cortex	5.4167	45	-71	-13	417.44	572	1.730	1.736	1.608
	R fusiform cortex	4.3463	40	-44	-27	452.93	828	1.929	1.830	1.486
	R posterior cingulate cortex	4.2717	4	-8	32	145.09	408	1.958	1.752	1.390
	R middle temporal cortex	4.2589	54	-4	-35	763.49	1164	1.830	1.781	1.594
	R posterior cingulate cortex	4.0841	8	-10	44	209.59	524	——	——	——
	R isthmus cingulate cortex	3.9943	6	-47	31	651.28	1511	2.197	2.004	1.735
	R superior parietal cortex	3.7380	28	-73	21	182.35	384	1.705	1.623	1.648
	R pars opercularis	3.7155	36	18	14	314.43	947	1.790	1.647	1.490
	R superior frontal cortex	3.7119	12	35	32	103.51	212	1.875	1.847	1.801
Mild < Moderate *p*-value <0.001	R superior frontal cortex	-3.7849	22	7	65	100.39	189	——	——	——
HCs > Moderate *p*-value <0.001 Surface area size > 100	L temporal pole	7.2720	-33	10	-39	340.30	691	——	——	——
	L insula	6.4471	-31	13	7	1720.84	4517	——	——	——
	L fusiform cortex	6.2268	-33	-34	-30	2075.62	3548	——	——	——
	L isthmus cingulate cortex	6.2197	-7	-39	35	5037.82	10788	——	——	——
	L lateral occipital cortex	5.1166	-44	-68	-8	1324.11	2433	1.707	1.743	1.607
	L middle temporal cortex	4.7842	-54	-10	-31	929.54	1306	1.813	1.809	1.595
	L inferior parietal cortex	4.6962	-32	-65	38	129.24	310	1.748	1.729	1.526
	L rostral middle frontal cortex	4.6635	-26	33	28	185.56	320	1.894	1.821	1.746
	L precentral cortex	4.4666	-41	1	27	295.74	703	1.592	1.624	1.630
	L inferior temporal gyrus	3.9811	-54	-39	-29	158.17	225	2.107	2.071	1.833
	L supramarginal gyrus	3.7352	-52	-43	31	145.87	329	1.590	1.602	1.464
	L superior parietal cortex	3.6853	-27	-77	16	290.15	533	1.683	1.653	1.660
	R precuneus	7.7128	10	-56	23	6221.94	13769	1.639	1.533	1.260
	R medial orbitofrontal	6.1465	11	19	-18	212.17	517	2.004	1.833	1.602
	R fusiform cortex	5.8971	30	-60	-17	2229.04	3877	——	——	——
	R postcentral gyrus	5.2073	42	-6	19	2128.36	5409	1.591	1.542	1.628
	R lateral orbitofrontal cortex	4.9392	29	31	-11	405.12	700	1.908	1.794	1.555
	R rostral middle frontal cortex	4.8654	28	50	1	228.63	363	1.853	1.780	1.733
	R middle temporal cortex	4.8144	54	-12	-31	1071.64	1639	——	——	——
	R superior parietal cortex	4.8007	30	-72	19	303.46	615	——	——	——
	R caudal middle frontal cortex	4.4205	34	4	33	156.69	340	1.707	1.684	1.680
	R inferior temporal gyrus	4.1356	51	-22	-35	176.07	277	——	——	——
	R cuneus cortex	4.0910	7	-83	13	256.64	333	1.656	1.522	1.462
	R precentral gyrus	3.7446	50	4	13	120.41	246	1.602	1.550	1.637
HCs > Mild *p*-value <0.01	L supramarginal gyrus	-3.2027	-54	-42	32	26.43	55	——	——	——

*PD patients with H-Y stage ≤1.5 were included in the mild group, and PD patients with 2.5≤ H-Y stage ≥2 were included in the moderate group. “——” means that more than one cluster located on the same brain areas, and these different clusters only have one mean cortical thickness. The *X*, *Y,* and *Z* coordinates refer to the anatomical location of brain areas*.

#### Cortical Thickness of the Moderate Group Compared With the Mild Group

**FIGURE 2:**
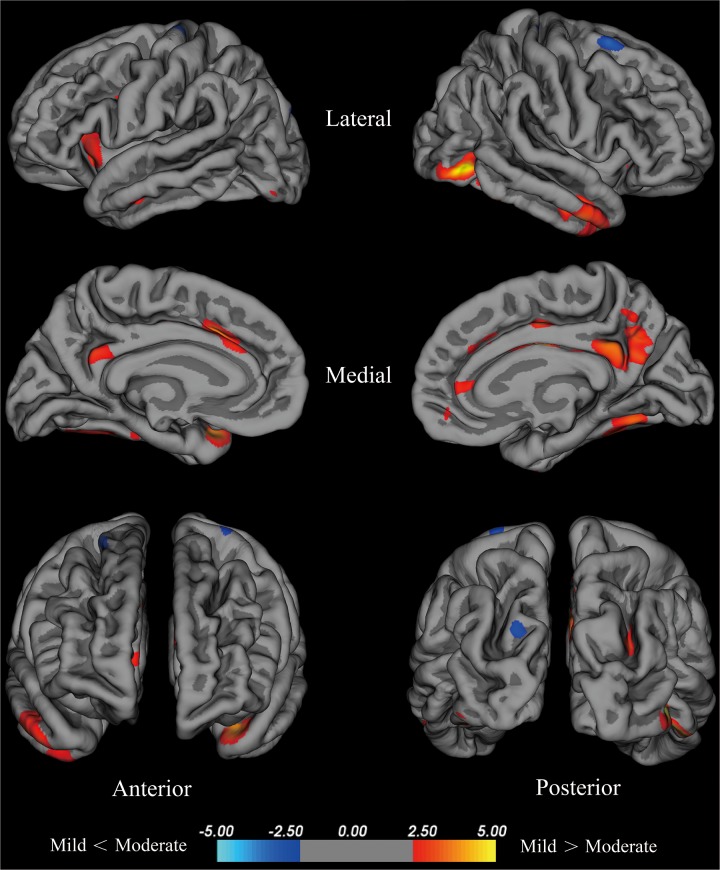
Brain areas with significant cortical thickness differences between the mild group and the moderate group in the right and left hemispheres (*p* < 0.001). PD patients with H-Y stage ≤1.5 were included in the mild group, and PD patients with 2.5≤ H-Y stage ≥2 were included in the moderate group. Red: mild > moderate, the closer the color is to yellow, the greater the difference. Blue: mild < moderate, the lighter the blue is, the larger the difference.

Compared with the mild group, the moderate group exhibited large areas (size > 100 mm^2^) of significant decreases (*p* < 0.001) in the temporal pole, superior frontal cortex, insula, isthmus cingulate cortex, fusiform gyrus of the left hemisphere, inferior temporal cortex, lateral occipital cortex, fusiform cortex, posterior cingulate cortex, middle temporal cortex, posterior cingulate cortex, isthmus cingulate cortex, superior parietal cortex, pars opercularis, and superior frontal cortex of the right hemisphere (Figure 2). Given that the cortical thickness different between these brain areas, these areas were selected to perform further analyses.

#### Cortical Thickness of the Moderate Group Compared With the HC Group

**FIGURE 3:**
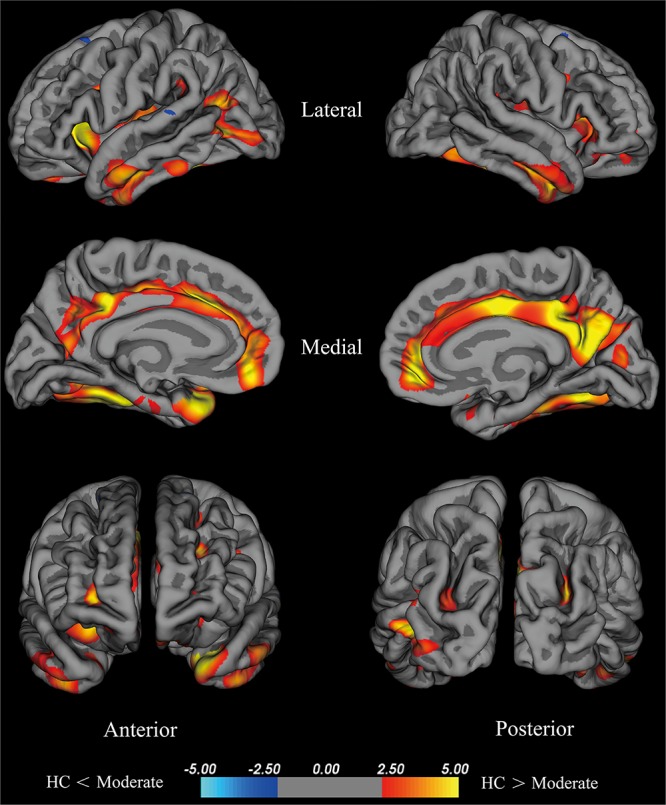
Brain areas with significant cortical thickness differences between the HC group and the moderate group in the right and left hemispheres (*p* < 0.001). PD patients with 2.5≤ H-Y stage ≥2 were included in the moderate group. Red: HC > moderate, the closer the color is to yellow, the greater the difference. Blue: HC < moderate, the lighter the blue is, the larger the difference.

The moderate group exhibited large areas (surface size > 100 mm^2^) of significant decreases (*p* < 0.001) in the temporal pole, insula, fusiform cortex, isthmus cingulate cortex, lateral occipital cortex, middle temporal cortex, inferior parietal cortex, rostral middle frontal cortex, precentral cortex, inferior temporal gyrus, supra marginal gyrus, and superior parietal cortex of the left hemisphere, precuneus, medial orbitofrontal, fusiform cortex, postcentral gyrus, lateral orbitofrontal cortex, rostral middle frontal cortex, middle temporal cortex, superior parietal cortex, and caudal middle frontal cortex of the right hemisphere (Figure 3). We hypothesize that these differential areas are related to PD.

#### Cortical Thickness of the Mild Group Compared With the HC Group

The mild group exhibited significant reductions in cortical thickness in the left supra marginal gyrus (*p* < 0.001).

#### Regression Analysis of Average Cortical Thickness

There were statistically significant differences in the average cortical thickness of the following eight brain areas: the temporal pole (*p* = 0.005), fusiform gyrus (*p* < 0.001), insula of the left hemisphere (*p* = 0.004), fusiform gyrus (*p* = 0.001), isthmus cingulate cortex (*p* = 0.001), inferior temporal gyrus (*p* = 0.003), middle temporal cortex (*p* = 0.001) and posterior cingulate cortex of the right hemisphere (*p* = 0.001) among the HC, mild and moderate groups. After controlling for age, gender, and duration, a regression analysis of the UPDRS-III scores of PD patients with the cortical thickness of the left and right fusiform gyrus and the left temporal pole was performed, results are shown in Table 3, the cortical thickness of these brain areas were negatively correlated with the UPDRS-III scores.

**Table 3:** Regression analysis of UPDRS-III scores and cortical thickness in different brain areas.

Regions	UPDRS-III		
	Adjusted *R*^2^	*p*-value	β
L fusiform gyrus	0.290	0.049	-0.227
L temporal pole gyrus	0.148	0.022	-0.291
R fusiform gyrus	0.285	0.039	-0.239

## Discussion

The current study analyzed regions of cortical thinning and comprehensively assessed the relationship between cortical thinning and UPDRS-III by comparing HC, mild PD and moderate PD groups. Our findings provide new insights into the pathogenesis and neuropathology of PD and may aid the development of new MRI biomarkers of disease progression.

Current limitations such as inadequate attention and difficulty diagnosing PD affect the treatment of the disease. Therefore, exploring the imaging features of PD patients can aid in the early diagnosis of PD. PD symptoms and disease severity are typically assessed using the UPDRS score and H-Y staging; H-Y staging ≤2.5 is considered to be the early stage of PD (Chen et al., 2016). Cortical atrophy, amygdalar atrophy and cortical thinning in the fronto-temporal region of PD patients were specifically associated with PD degeneration (Ibarretxe-Bilbao et al., 2012). Age (Uribe et al., 2016), gender (Luders et al., 2006), disease duration (Madhyastha et al., 2015) are associated with the cortical thickness, which might reflect a mixed pathologic features, such as neuronal density (La Fougere et al., 2011), cerebral perfusion (Nobili et al., 2011; Madhyastha et al., 2015), degenerative pathologies including tau, α-synuclein in PD patients (Ye et al., 2017). Deng et al. (2016b) found that the cortical morphological changes of brain regions (i.e., frontal, temporal, parietal, occipital and limbic lobes; cerebellum; caudate; and thalamus) were closely related to clinical symptoms of sporadic PD patients, including H-Y stage and UPDRS-III scores. This finding is consistent with our finding that the PD patients exhibited significantly lower mean cortical thickness of the temporal pole, fusiform gyrus, insula of the left hemisphere and the fusiform gyrus, isthmus cingulate cortex, inferior temporal gyrus, middle temporal cortex and posterior cingulate cortex of the right hemisphere than the HC group. In addition, UPDRS-III scores in PD patients were negatively correlated with the left and right fusiform gyrus and left temporal pole, thus providing further evidence of MRI biomarkers for specific brain changes in the pathophysiology of PD motor dysfunction.

Many different methods have been used in early PD cross-sectional imaging studies, and there are large variations in the structural changes of the cortex and WM. For example, changes in cortical thickness (Mak et al., 2015), GM volume (Jia et al., 2015) and cortical microstructure (Nurnberger et al., 2017), increased cortical and subcortical atrophy rate (Tessa et al., 2014) and loss of cortical gyrification (Sterling et al., 2016) have been reported, and some imaging signs even correlate with disease progression (Ibarretxe-Bilbao et al., 2011; Jubault et al., 2011). Another previously published study reported that disease stage in PD was associated with thinning of the medial frontal, posterior cingulate, precuneus, lateral occipital, temporal and dorsolateral prefrontal cortex (Zarei et al., 2013). In contrast to our study, which enrolled PD patients with H-Y stage ≤2.5, Zarei et al. divided PD patients into three groups: group 1 consisted of PD patients with H-Y stage ≤2, group 2 consisted of PD patients with H-Y stage 2–4, and group 3 consisted of PD patients with H-Y stage >3 (Zarei et al., 2013). In our study, the temporal cortex (including the temporal pole, fusiform gyrus of the left hemisphere, fusiform gyrus, inferior temporal gyrus, and middle temporal cortex of the right hemisphere), and the posterior cingulate cortex of the right hemisphere were consistent with Zarei et al.’s finding (Zarei et al., 2013). A pattern of GM changes has been suggested to occur in the mesial frontal cortex in PD patients with FOG. Pietracupa et al. (2017) found that PD patients with FOG exhibit significant changes in WM and cortical thinning of the mesial surface of the bilateral cerebral hemispheres. Vastik et al. (2017) found that FOG was associated with regional GM atrophy in the mesial frontal and cingulate cortices. Consistent with our results, the thickness of the posterior cingulate cortex and the temporal cortex were correlated with H-Y stages. Previous research demonstrated that mirror movements were associated with bilateral overactivation of the precuneus/posterior cingulate cortex and the insula (Harel et al., 2013). Another study revealed that cortical thickness changes in the bilateral posterior cingulate cortex were correlated with a higher cognitive risk score (Ye et al., 2017). The results point to the likelihood that a decline in regional cerebral blood flow (rCBF) in the posterior cingulate cortex is related to PD disease progression (Imamura et al., 2008; Nobili et al., 2011). Recent studies have found that decreased cortical thickness of the fusiform gyrus is related to cognitive impairment (Pagonabarraga et al., 2013; Zarei et al., 2013). Interestingly, temporal pole atrophy is consistent with the Braak classification of PD pathology (Potgieser et al., 2014). We believe that the combination of different MRI techniques and brain areas may contribute to improving evaluations of progressive brain involvement and may eventually reveal surrogate markers of disease progression.

Given that different H-Y stages indicate different disease progression stages of PD, different H-Y stages exhibit different neuropathology, neuroimaging, and/or clinical evidence. Thus, imaging studies focusing on PD patients at different H-Y stages can help understand the pathogenesis and disease progression of PD. NMS are extremely common throughout all stages of PD and are more critical to detect in the early stages of the disease, as NMS are closely related to the pathological process of PD. In recent years, the value of recognizing NMS of PD for the early diagnosis of PD has been noted (Berg et al., 2015; Postuma et al., 2015). Therefore, better identifying NMS may increase the possibility of detecting PD biomarkers in the early stage of PD, and the development of novel MRI methods will provide further new imaging markers for the early diagnosis and treatment of PD. In a study of mid-sporadic PD patients, Deng et al. (2016a) reported that dysfunctions in some brain areas with cortical thinning are closely related to clinical symptoms, such as cognitive impairment, hallucinations, psychosis, depressed mood, anxious mood, apathy, sleep problems, and sexual desire disorder. Other studies have reported abnormal changes, such as cortical thinning (Danti et al., 2015; Uribe et al., 2016) and GM volume atrophy (Foo et al., 2017), in PD patients with cognitive impairment, whereas PD patients with RBD (Boucetta et al., 2016), depression (Huang et al., 2016; Luo et al., 2016), impulsivity (Pellicano et al., 2015) and NMS also exhibit cortical changes. Many NMS during the PD prodromal phase are clearly associated with the occurrence of PD and are clear risk factors. Therefore, cortical changes in PD patients associated with NMS have an important role in the early diagnosis of PD.

Numerous studies have confirmed that cognitive dysfunction and motor symptoms are correlated. In newly diagnosed PD patients, the severity of motor symptoms at the first visit correlates with cognitive impairment and is associated with an increased risk of developing MCI (Siciliano et al., 2017). The postural instability gait difficulty motor phenotype is a risk factor for the development of PD dementia (Muller et al., 2013). Dysfunction in the right dorsal medulla oblongata shell and core can account for association learning deficits in patients with left-sided PD (Huang et al., 2017). Other studies confirmed that progression on the H-Y scale positively predicted the extent of cognitive impairment (Wu et al., 2012; Pedersen et al., 2013). However, other reports suggest no correlation between cognition and motor function. For example, Weintraub et al. (2015) did not identify a significant correlation between H-Y classification and cognitive impairment using the MoCA scale, but the study was limited by a lack of effective neurocognitive scales. The fronto-temporo-parietal pattern of cortical thinning pattern does not associate with motor laterality (Danti et al., 2015). In the prodromal stage of PD, a progressive process could occur from non-motor to motor performances until the disease becomes clinically evident, which is consistent with Braak’s proposed mechanism. Therefore, PD imaging studies should be combined with studies on motor and NMS, especially RBD and cognitive dysfunction, to contribute to the diagnosis of early and even prodromal PD.

The main limitations of this study include three points. First, the sample size included in this study is relatively small, and we need to increase the sample size and conduct follow-up observations in subsequent studies. Second, further studies are needed to quantify potential differences between PD patients with H-Y ≤2.5 and those with H-Y >2.5. Third, the study controlled for age, gender and duration to avoid confounding effects, but there may be confounding factors (e.g., years of education and cognitive score) that should be addressed in future studies.

In conclusion, the HC, mild and moderate groups exhibited significant decreasing trends in the mean cortical thickness of the following eight brain areas: the temporal pole, fusiform gyrus, insula of the left hemisphere and fusiform gyrus, isthmus cingulate cortex, inferior temporal gyrus, middle temporal cortex and posterior cingulate cortex of the right hemisphere. The UPDRS-III scores in patients with early PD were correlated with cortical thickness of the left and right fusiform gyrus and left temporal pole. The results of this study provide a research direction for basic research aimed to better understand neuropathology in PD patients, contribute to the early diagnosis of PD, and provide a new neuroimaging biomarker for PD.

## Author Contributions

YG, KN, YZ, and LjW conceived and designed the study and drafted the manuscript. BH is responsible for performing MRI studies. MM is responsible for performing MRI data processing. MG is helped draft the manuscript. YG, ZH, LmW, and JZ conceived and coordinated the neurocognitive evaluation and helped with statistical analysis. YZ and LjW helped draft the manuscript and supervised the research project. All authors read and approved the final manuscript.

## Conflict of Interest Statement

The authors declare that the research was conducted in the absence of any commercial or financial relationships that could be construed as a potential conflict of interest.

## Footnotes

1 http://surfer.nmr.mgh.harvard.edu

## Abbreviations

AD: Alzheimer’s disease
ADL: activities of daily living
CT: computed tomography
DTI: diffusion tensor imaging
FOG: freezing of gait
GM: gray matter
HAMA: Hamilton Anxiety Scale
HAMD: Hamilton Depression Scale
HC: healthy control
H-Y stage: Hoehn and Yahr stage
MDS: movement disorder society
MMSE: mini mental state examination
MoCA: montreal cognitive assessment
MRI: magnetic resonance imaging
NMS: non-motor symptoms
PD: Parkinson’s disease
PDD: Parkinson’s disease with dementia
PD-MCI: Parkinson’s disease with mild cognitive impairment
PD-NC: Parkinson’s disease with normal cognition
UPDRS-III: part III of the Unified Parkinson’s Disease Rating Scale
WM: white matter
